# Dataset of Indian and Thai banknotes with annotations

**DOI:** 10.1016/j.dib.2022.108007

**Published:** 2022-03-02

**Authors:** Vidula Meshram, Kailas Patil, Prawit Chumchu

**Affiliations:** a Vishwakarma University, India; b Kasetsart University, Thailand

**Keywords:** Banknote recognition, Counterfeit banknote, Currency detection, Indian banknotes, Thai banknotes, Machine learning

## Abstract

Multinational banknote detection in real time environment is the open research problem for the research community. Several studies have been conducted for providing solution for fast and accurate recognition of banknotes, detection of counterfeit banknotes, and identification of damaged banknotes. The State-of art techniques like machine learning (ML) and deep learning (DL) are dominating the traditional methods of digital image processing technique used for banknote classification. The success of the ML or DL projects heavily depends on size and comprehensiveness of dataset used. The available datasets have the following limitations:

1. The size of existing Indian dataset is insufficient to train ML or DL projects [1], [2].

2. The existing dataset fail to cover all denomination classes [1].

3. The existing dataset does not consists of latest denomination [3].

4. As per the literature survey there is no public open access dataset is available for Thai banknotes.

To overcome all these limitations we have created a total 3000 image dataset of Indian and Thai banknotes which include 2000 images of Indian banknotes and 1000 images of Thai banknotes. Indian banknotes consist of old and new banknotes of 10, 20, 50, 100, 200, 500 and 2000 rupees and Thai banknotes consist of 20, 50, 100, 500 and 1000 Baht.

## Specifications Table

**Table:** 

Subject	Machine Learning
Specific subject area	Banknote detection and identification
Type of data	Indian and Thai banknote images
How data were acquired	The Indian and Thai banknote images were collected by taking their images using high resolution mobile phone camera.
Data format	Raw Annotated
Parameters for data collection	The Indian and Thai Banknote dataset images are .jpg images of 1024 × 1024 dimension and resolution is 96 dpi
Description of data collection	The banknote images of India and Thailand were collected using high resolution mobile phone camera. The original .jpg images of banknotes are of dimensions 3024 × 3024. These images are resized to 1024 × 1024 dimension. There are total 10 classes of Indian banknotes namely 10, 20, 50, 100, 200, 500 and 2000 rupees and 5 classes of Thai banknotes 20, 50, 100, 500 and 1000 Baht.The banknote images were taken in various environmental conditions like dark back ground, illuminated background, cluttered environment, occluded banknote, folded banknote, inside the lab outside the lab.
Data source location	VISHWAKARMA UNIVERSITY Survey No. 2, 3, 4 Laxmi Nagar, Kondhwa Budruk, Pune - 411 048. Maharashtra, India.
Data accessibility	Repository name: Dataset of Indian (Rupees) and Thai (Baht) Banknotes with Annotations Data identification number(doi): 10.17632/2kfz5yc7pt.1 Direct URL to data: https://data.mendeley.com/datasets/2kfz5yc7pt/1

## Value of the Data

• The dataset is comprehensive which consist of 3000 high-quality images of 15 different classes.
• The dataset consist of old as well as new denominations of Indian banknotes.
• This dataset is useful to build applications of Indian and Thai banknotes classification and detection. It can also be used by researchers working in domain of banknote classification and identification.
• This dataset is useful for training, testing and validation of Indian or Thai banknotes it or for both classification and identification models.
• The dataset will play an important role for value identification of both India and Thai banknotes.
• The dataset is useful to build application for banknote classification, identification, and detection which can be used by visually impaired people, bank customers and governments.

## 1 Data Description

**Fig. 1:**
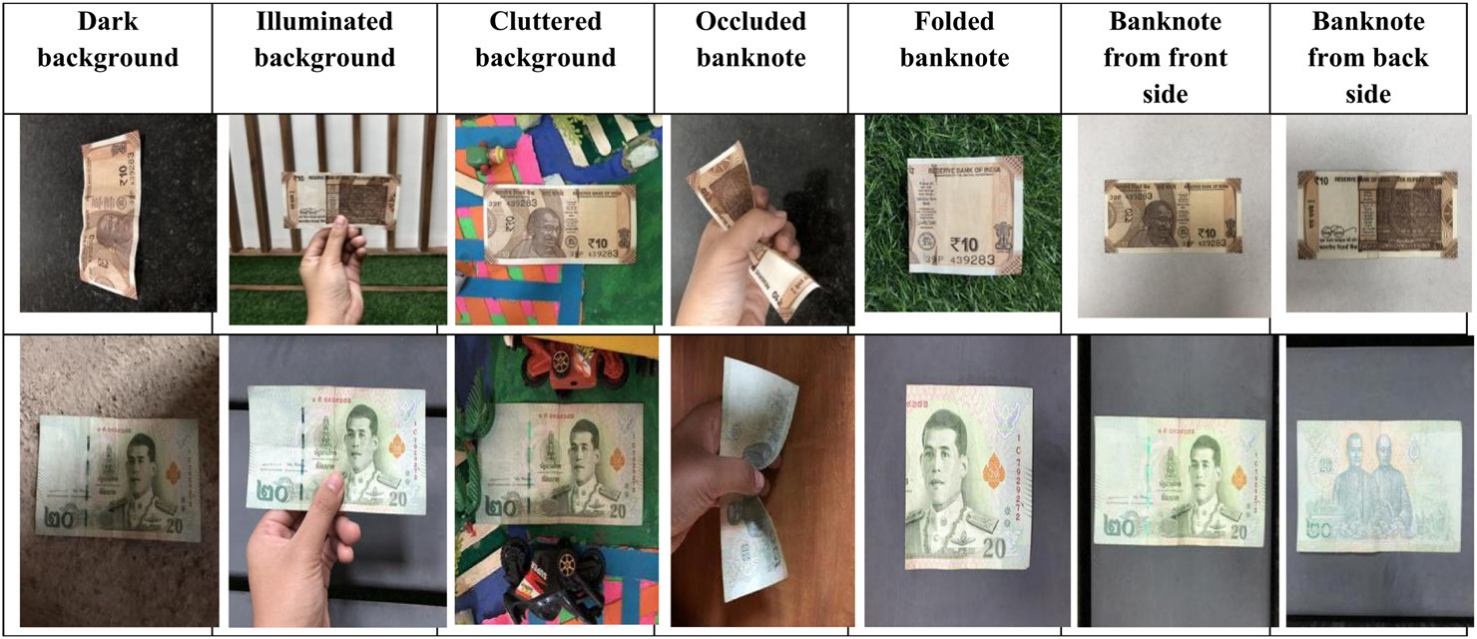
Banknote images taken in various environments.

The development of banknote dataset is very crucial due to following reasons, Correct recognition of banknotes is very important task for automated teller machines and currency recognition machines [4], it is also necessary to develop a system which detects the banknote is genuine [5], further recognition of banknotes is one of the problems faced by visually impaired people [6], [7]. Our banknote dataset is collection of Indian and Thai banknotes. It consist of 10 classes of Indian banknotes namely 10, 20, 50, 100, 200, 500 and 2000 Rupees and 5 classes of Thai banknotes 20, 50, 100, 500 and 1000 Baht. The banknote image dataset was taken in various environmental conditions like in illuminated environment, dark environment, in cluttered background. Also images of half folded or occluded banknote images are taken. Hence this dataset will be very helpful to researchers for performing experiments such as recognition and classification of banknotes. The Fig. 1 shows the sample images in the dataset consisting of images taken in various environments.

The Indian and Thai bank note images and its annotations in text format are stored in Indian_ Thai_BankNotes_Dataset folder which is the main folder. This main folder consist of two subfolders namely IndianBankNotes and ThaiBanknotes, which in turn consists of subfolders training and validation. The subfolder training and validation consists of image and its annotated file in YOLO format. The annotation labels for India banknotes were done by considering respective denominations and image of Mahatma Gandhi on banknotes. The annotation labels for Thai banknotes were done by considering respective denominations and image of King Rama on the banknote. The YOLO format allow us to save annotations in txt files in following format:

<class> <x-min>, < y-min>, <width >, <height>

**Fig. 2:**
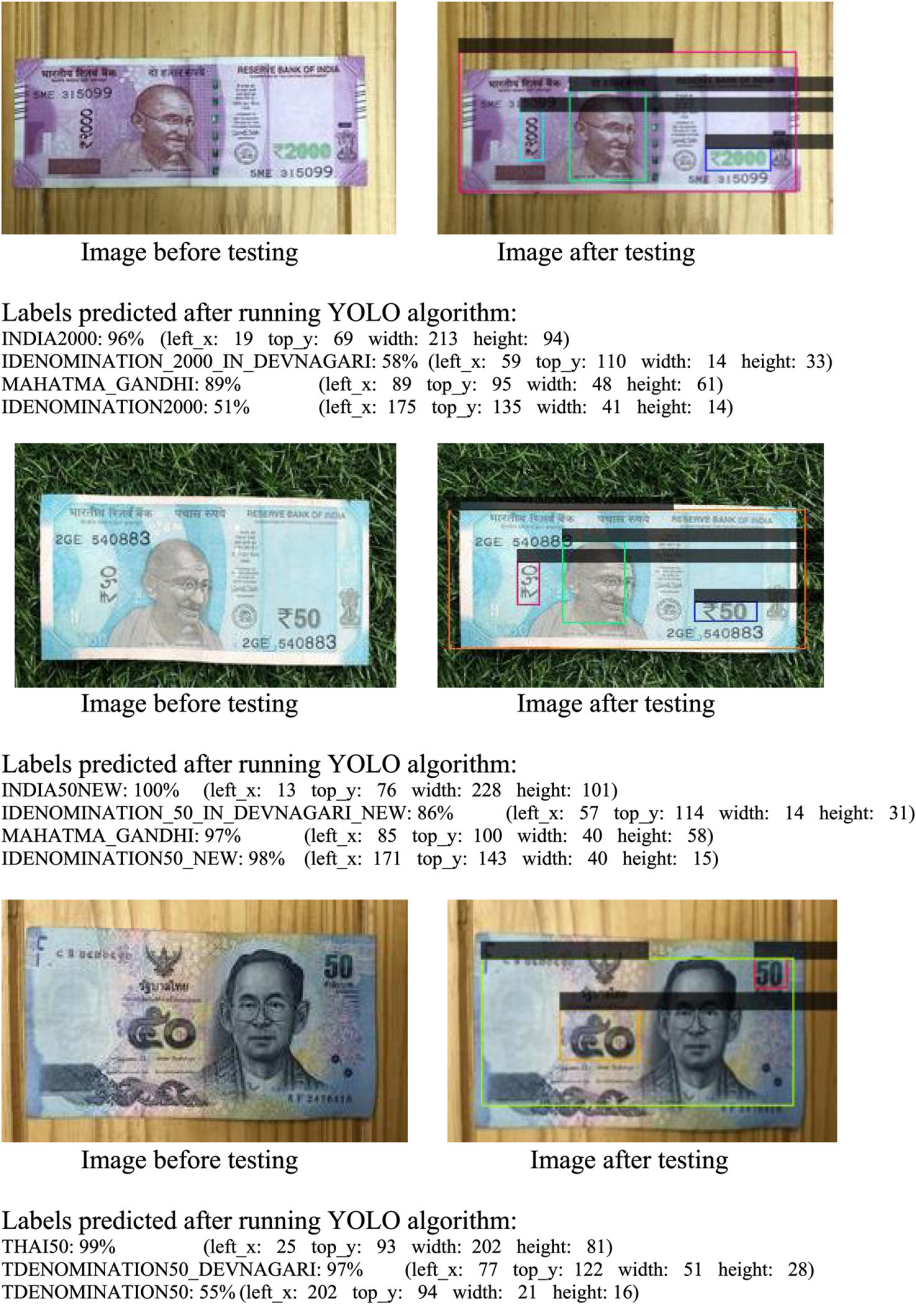
Labeled banknote images after running YOLO algorithm.

**Fig. 3:**
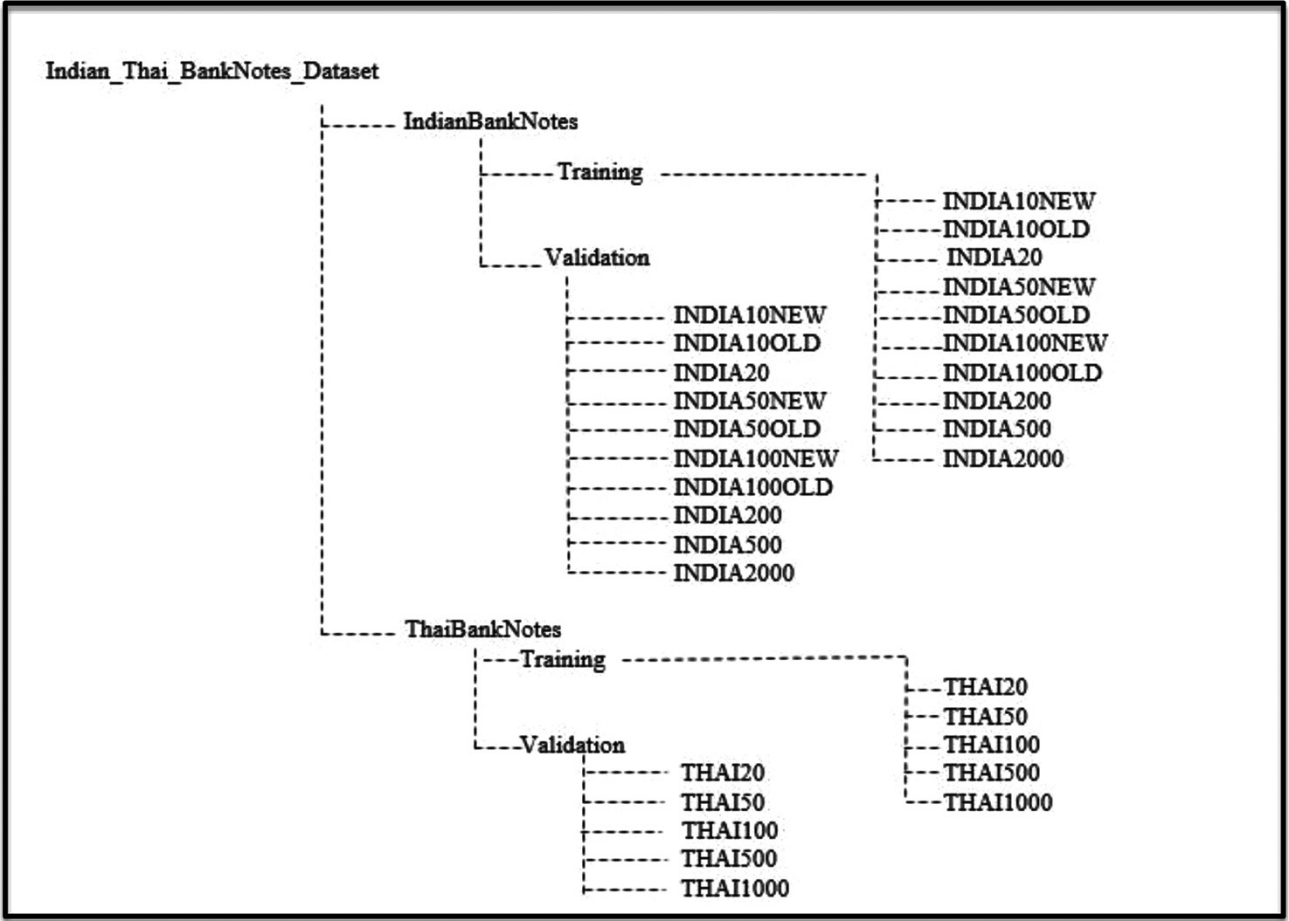
Banknote dataset directory structure.

The image before testing and its labeled image after running YOLO algorithm are shown in Fig. 2 and the directory structure of banknote dataset is as shown in Fig. 3.

## 2 Experimental Design, Materials and Methods

### 2.1 Experimental design

**Fig. 4:**
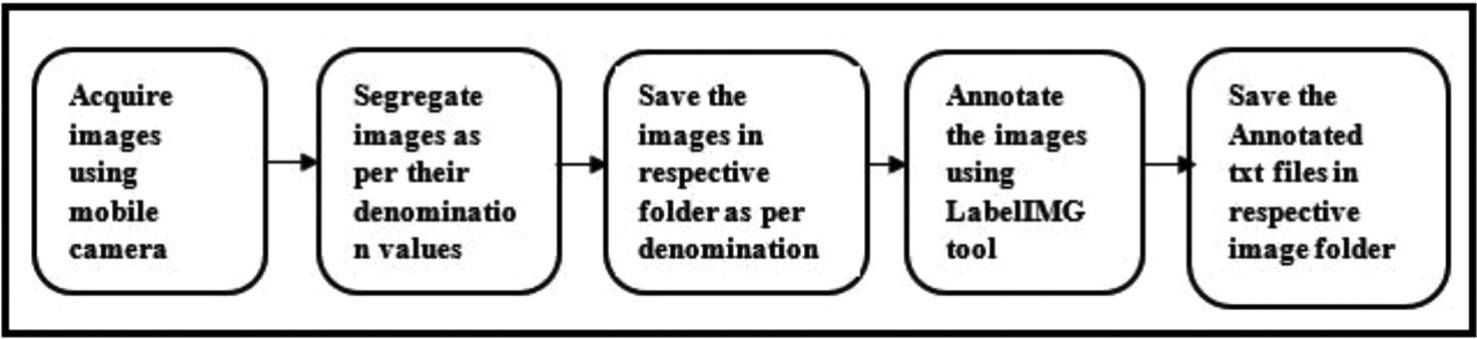
Banknote data acquisition process.

The image data acquisition process is shown in Fig. 4. The banknote images were acquired using iPhone8 mobile's high resolution rear camera. In all 3000 (2000 Indian + 1000 Thai) images were captured using camera and then were segregated and saved in respective folders as per their denomination values.

The data acquisition process steps are shown in Table 1 and the details of image acquisition is specified in Table 2. The banknote images are captured daily during daytime in months of July to August using iphone8 rear camera. The images are captured in different directions and backgrounds as mentioned in Table 3. After capturing images of both Indian and Thai bank notes, the images were segregated in proper folders. The detail folder structure of images is shown in Fig. 3. The banknote images resized using python script. Then the images were annotated using LabelImg tool from the month of September to November.

**Table 1:** Data acquisition steps.

Sr. No.	Step	Duration	Activity
1.	Data Gathering	July to August	Daily during daytime captured the banknote images
2.	Image Labeling	September to November	Labeled the 2000 images of Indian banknotes and 1000 images of Thai banknotes

**Table 2:** Specification of image acquisition.

Sr. No.	Particulars	Details
1	Camera	(i) Make and Model: Apple iPhone8
		(ii) Rear Camera: 12-megapixel (f/1.8)
		(iii) Rear autofocus
2	Battery	1821 mAh
3	Labeling Software	LabelImg
4	Image Resolution	1024 × 1024
5	Image Format	JPG

**Table 3:** Banknote details.

Banknotes	Denominations Considered	Direction of image Capturing	Different Backgrounds considered for image capturing	No. of Images of each denomination	Total No. of Images
India	10 New and Old, 20 New and Old, 50 New and Old, 100, 200, 500, 2000 Rupees	Front Direction, Front Direction Rotated 180°, Backward Direction, Backward Direction Rotated 180°, Half folded	Illuminated, Dark, cluttered, Occluded	200	2000
Thai	20, 50, 100, 500, 1000 Baht	Front Direction, Front Direction Rotated 180°, Backward Direction, Backward Direction Rotated 180°, Half folded	Illuminated, Dark, cluttered, Occluded	200	1000

### 2.2 Materials or specification of image acquisition system

The banknote images are captured using Apple iphone8 with rear camera of 12 MP. All dataset images of original size 3024 × 3024 were resized to 1024 × 1024 dimension using a python script. The images are saved in .jpg format. The images are captured in variety of environmental conditions such as different light conditions, different background, from different angles, folded and occluded banknote situations.

After capturing the images they were organized as Indian banknotes and Thai Banknotes. The Indian banknotes consist of 10 different folders of respective Indian banknotes and the Thai banknotes consist of 5 different folders of respective Thai banknotes. The dataset directory structure of images is shown in Fig. 3. The images are annotated using LabelImg tool. The annotations and images of banknote are stored in their respective folders.

### 2.3 Method

All banknote images are acquired using iphone 8 mobile rear camera in different angles and different backgrounds. The orignal images of size 3024 × 3024 were resized to 1024 × 1024 using a python script and then labeled using LabelImg tool Table 3. describes the classes, number of image taken and the environments in which images are taken.

## Ethics Statement

There is no funding present for present effort. There is no conflict of interest. The data is available in public domain.

## CRediT authorship contribution statement

**Vidula Meshram:** Methodology, Software, Data curation, Writing – original draft. **Kailas Patil:** Conceptualization, Supervision, Writing – review & editing. **Prawit Chumchu:** Supervision.

## Declaration of Competing Interest

The authors declare that they have no known competing financial interests or personal relationships that could have appeared to influence the work reported in this paper.

## Appendix Supplementary materials

Image, application 1
